# Community pharmacies in early detection of obstructive sleep apnea: findings from a nationwide survey

**DOI:** 10.3389/fpubh.2025.1712922

**Published:** 2025-11-28

**Authors:** Francesco Giombi, Luca Cerri, Michele Cerasuolo, Gian Marco Pace, Giulio Sandri, Alberto Braghiroli, Giuseppe Mercante, Giuseppe Spriano, Michele Cucchi, Stefano Aliberti, Enrico Heffler, Walter Canonica, Giovanni Paoletti, Enrico Keber, Corrado Giua, SIFAC Group, Luca Malvezzi

**Affiliations:** 1 Otorhinolaryngology-Head and Neck Surgery Unit, Casa di Cura Humanitas San Pio X, Milan, Italy; 2 Otorhinolaryngology-Head and Neck Surgery Unit, IRCCS Humanitas Research Hospital, Milan, Italy; 3 Department of Biomedical Sciences, Humanitas University, Milan, Italy; 4 Sleep Care Center, Humanitas Mater Domini Hospital, Castellanza, Italy; 5 Medical Care Division, Head of Clinical Product and Solution, Milan, Italy; 6 Respiratory Unit, IRCCS Humanitas Research Hospital, Milan, Italy; 7 Personalized Medicine, Asthma and Allergy, IRCCS Humanitas Research Hospital, Milan, Italy; 8 Società Italiana Farmacia Clinica (SIFAC), Cagliari, Italy

**Keywords:** obstructive sleep apnea, sleep-disordered breathing, Berlin questionnaire, sleep dysfunction, Pittsburgh sleep quality index, community pharmacy

## Abstract

**Introduction:**

Obstructive sleep apnea (OSA) is a highly prevalent, yet underdiagnosed sleep disorder associated with cardiovascular, metabolic, and neurocognitive morbidity, as well as impaired quality of life. Limited access to diagnostics, low public awareness, and underreporting of symptoms contribute to a substantial gap in detection. Community pharmacies, given their accessibility and frequent interaction with patients managing multiple comorbidities, represent a promising but underexplored setting for identifying individuals at high risk.

**Objective:**

To investigate the feasibility of pharmacy-based screening for OSA and to provide preliminary insights into the prevalence of at-risk individuals among pharmacy clients.

**Methods:**

A cross-sectional study was conducted in 22 Italian community pharmacies, where participants completed a three-section questionnaire recording demographic data, the Berlin Questionnaire (BQ) for OSA screening and the Pittsburgh Sleep Quality Index (PSQI) for sleep quality assessment. Multivariate regression was performed to explore the association between poor questionnaire outcomes, demographic variables, and ongoing medication use. A sensitivity analysis was conducted to minimize the risk of bias.

**Results:**

A total of 301 individuals were included (females: *n* = 169/301, 56.15%). One-hundred-sixteen subjects (38.5%, *n* = 301) scored positively in at least two categories of the BQ and were hence classified as at-risk. At sensitivity analysis, BMI (OR = 1.15, 95%CI: 1.07–1.24, *p* < 0.001), and ongoing antihypertensive medications (OR = 2.02, 95%CI: 1.78–3.11, *p* = 0.002) were associated with poor BQ outcome. A significantly higher PSQI score was observed compared to previously reported values in healthy individuals. However, no significant associations were observed between poor sleep quality and patients’ demographics, or ongoing medication use.

**Conclusion:**

Community pharmacies can serve as a valuable setting for the early identification of individuals at risk for sleep-related breathing disorders, particularly among patients with multiple comorbidities. By leveraging their accessibility and frequent patient contact, pharmacies may complement existing healthcare pathways and support efforts aimed at reducing the current diagnostic gap in OSA.

## Introduction

Obstructive sleep apnea (OSA) is a common and increasingly recognized sleep disorder characterized by frequent episodes of upper aerodigestive tract obstruction during sleep, leading to intermittent hypoxia, sleep fragmentation, and a wide range of health complications (1). Affecting upwards of 1 billion individuals globally, according to some estimates, OSA has become a significant public health concern due to its association with cardiovascular diseases, metabolic disorders, neurocognitive impairments, and overall reduced quality of life (2–5). Despite significant advancements in understanding its pathophysiology and diagnosis, the true burden of OSA in the general population remains underestimated, as most cases remain undiagnosed in both developing and developed countries of the world (2).

Epidemiological studies have consistently showed that OSA is highly prevalent, with varying rates influenced by demographic, geographic, and methodological factors (6). Prevalence estimates range widely, from 14–49.7% in men and 3.7–23.4% in women, with higher rates among older and obese individuals (7–10).

Despite its high prevalence and significant health implications, OSA remains underrecognized due to diagnostic barriers, including limited access to sleep medicine services, lack of awareness among healthcare providers and the public, and the underreporting of symptoms such as snoring and daytime sleepiness.

In this context, community pharmacists may play a pivotal role in the early identification of individuals at risk for obstructive sleep apnea (11). As highly accessible healthcare professionals, pharmacists operate at the front line of patient interaction, particularly among individuals with multiple comorbidities and polypharmacy, who are more likely to exhibit risk factors for sleep disordered breathing (12).

This nationwide survey, carried out across community pharmacies throughout Italy, was therefore conducted to investigate the feasibility of pharmacy-based screening for OSA and to provide preliminary insights into the prevalence of at-risk individuals among pharmacy clients.

## Materials and methods

### Study design

This is a cross-sectional study based on a three-part questionnaire, conducted as exempt research in compliance with the ethical standards of the Declaration of Helsinki and its later amendments. The project was developed in collaboration between a team of researchers from the Italian Society of Clinical Pharmacy (SIFAC) and the Department of Otorhinolaryngology—Head and Neck Surgery of Casa di Cura Humanitas San Pio X in Milan, Italy. The survey consisted of three distinct sections: The first contained items pertaining to the self-reported general health status of the patient, investigating information such as age, sex, tobacco and alcohol use, anthropometric data such as weight and height, and ongoing pharmacological therapy (including anti-hypertensive medication, anti-arrhythmic medication, diabetes medication, and 5-phosphodiesterase inhibitors); the second and third sections of the survey consisted in the completion of two questionnaires: the Berlin questionnaire (13), a screening tool to assess the risk of obstructive sleep apnea, and the Pittsburgh Sleep Quality Index (14), aimed at exploring general subjective sleep dysfunction.

The three-part survey was homogeneously administered by clinical pharmacists working in 22 community pharmacies evenly distributed across the Italian territory. Data collection took place between October and December 2024 through a Google Form tool and was subsequently analyzed in an anonymized format. Each participant signed an informed consent for the data collection and analysis.

Patients who met at least one of the following criteria were deemed eligible for enrollment: (i) currently being under antiarrhythmic, antihypertensive, 5-phosphodiesterase inhibitor, or diabetes medications; (ii) requiring prescription treatment or over-the-counter drugs for insomnia; (iii) seeking advice for snoring or improvement of sleep quality. Those who were already diagnosed with sleep-related breathing disorders or under treatment with continuous-positive airway pressure (c-PAP) were excluded from the analysis. Likewise, minors and individuals unable to independently complete the questionnaire were excluded.

### Questionnaires

The Berlin Questionnaire (BQ) consists of three different categories and aims to assess the risk of obstructive sleep apnea. Based on their responses to individual items and their cumulative scores within these symptom categories, patients are classified as either high-risk or low-risk (13). Category 1, consisting of five items, focuses on snoring behaviors. Category 2, with its three items, investigates daytime sleepiness. Category 3 includes a single item that inquiries about the presence of hypertension. A positive score in the first two categories requires frequent symptom occurrence, defined as more than 3–4 times per week. In contrast, a positive score in the third category results from either a history of hypertension or a BMI greater than 30 kg/m^2^. Patients are classified as high-risk if they score positively in at least two categories; otherwise, they are considered low-risk.

The second questionnaire, the Pittsburgh Sleep Quality Index (PSQI), consists of 19 self-reported items grouped into seven sleep domains: subjective sleep quality, sleep latency, sleep duration, habitual sleep efficiency, sleep disturbances, use of sleep medications, and daytime dysfunction (14). Participants completed the questionnaire based on their sleep patterns over the preceding month. Each item is scored from 0 to 3, with the total scores of the seven components contributing to the global PSQI score (range: 0–21). A global score over 5 indicates poor sleep quality. The validated Italian translations of the questionnaires were used (15). The full survey is available as Supplementary Content 1.

### Data analysis

Anonymized data were collected at the conclusion of the survey and summarized using descriptive statistics. Categorical variables were classified by counts and percentages, while continuous variables were reported as range and mean ± standard deviation (SD). The sample size was calculated assuming a 95% confidence level and an expected proportion of high-risk patients of 32.6%, based on previously reported prevalence data from BQ outcomes in the general population (16). For this estimated prevalence, the margin of error was set at 5.3%. Based on these assumptions, the required sample size was calculated to include 301 subjects. Data analysis was conducted with IBM® SPSS Software for Macintosh, Version 26.0. Statistical significance was defined as *p* < 0.05. Difference between categorical variables was assessed using *chi-square (χ^2^)* test. Normal distribution of included variables was confirmed through *Shapiro–Wilk* test (*p*-value > 0.05). Continuous parametrical variables were compared using *Student t-test* for unpaired samples. Binary logistic regression analysis was used to measure the risk for questionnaires’ unfavorable outcome based on dichotomous baseline characteristics. A multivariate logistic regression model was used to minimize the risk of confounding factors. The strength of the association was expressed in terms of Odds Ratio (OR) and 95% Confidence Intervals (CI). To mitigate the risk of circularity, a sensitivity analysis was performed excluding patients who were referring to the pharmacies for snoring or sleep-quality concerns, as well as those seeking advice for insomnia. The Variance Inflation Factor (VIF) was used to assess multicollinearity, which reflects the degree of intercorrelation among the variables included in the regression model and the potential bias it may introduce. According to established criteria, a VIF value < 3 was considered indicative of acceptable multicollinearity (17). The assumption of linearity of the logit for continuous variables was verified using the Box–Tidwell transformation and confirmed for a *p*-value > 0.05.

## Results

### General characteristics

**Figure 1:**
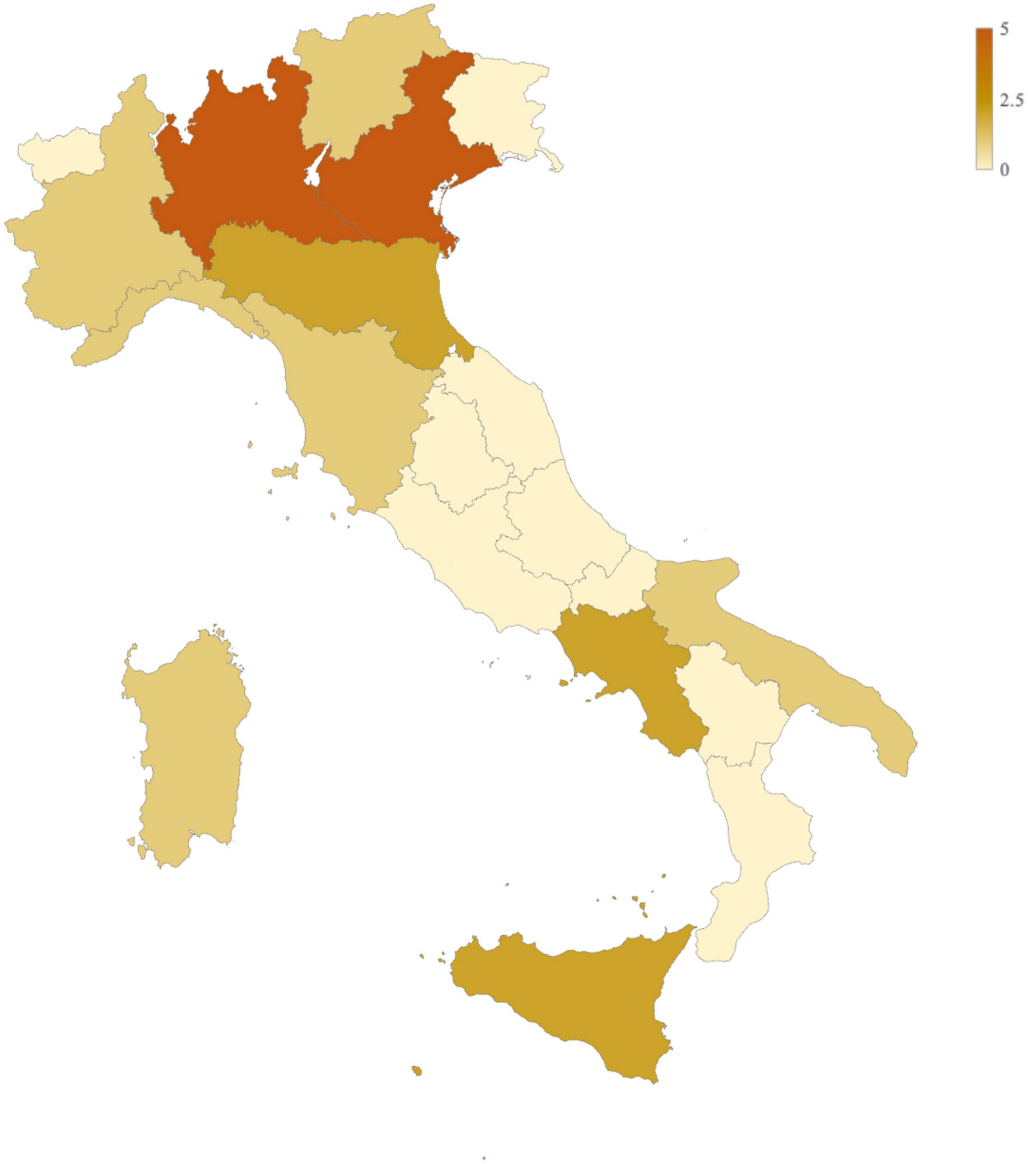
Included pharmacies by region.

A total of 22 pharmacies across 11 Italian regions participated in the survey (Figure 1). The majority were located in northern Italy, including Lombardy (*n* = 5/22, 22.73%), Veneto (*n* = 5/22, 22.73%), Piedmont (*n* = 1/22, 4.55%), Trentino-Alto Adige (*n* = 1/22, 4.55%), and Liguria (*n* = 1/22, 4.55%). Pharmacies from central (Emilia-Romagna: *n* = 2/22, 9.09%; Tuscany: *n* = 1/22, 4.55%) and southern regions (Puglia: *n* = 1/22, 4.55%; Campania: *n* = 2/22, 9.09%; Sardinia: *n* = 1/22, 4.55%; Sicily: *n* = 2/22, 9.09%) were also included, achieving broad geographical representation.

In total, 301 individuals met the inclusion criteria and were included in statistical analysis. On average, each pharmacy enrolled 13.7 individuals, with a homogeneous normal distribution (range: 7–20, SD: 2.98, Shapiro–Wilk test: *p*-value = 0.568). The cohort consisted of 132 men (43.85%) and 169 women (56.15%), with a mean age of 56.36 years (range: 23–91; SD = 14.7). The mean BMI was 26.07 (range: 16.2–47.8; SD = 4.82). A total of 79 participants (26.25%) were smokers, while 222 (73.75%) had never smoked.

Regarding alcohol consumption, 91 participants (30.23%) reported never drinking, 82 (27.24%) consumed alcohol less than once per week, 85 (28.24%) drank once or twice per week, 19 (6.31%) drank three to four times per week, and 24 (7.97%) consumed alcohol daily.

As for medication use, 151 participants (50.17%) were taking antihypertensive medications, 15 (4.98%) were using phosphodiesterase-5 (PDE5) inhibitors, 65 (21.59%) were on antiarrhythmic medications, and 55 (18.27%) were receiving antidiabetic treatment.

### Questionnaires

#### Berlin questionnaire

Results from single categories of the BQ in the overall population are presented in Table 1.

**Table 1:** Results from single categories of the Berlin questionnaire.

	Category 1	Category 2	Category 3
Positive (%)	156 (51.8)	73 (24.3)	147 (48.8)
Negative (%)	145 (48.2)	228 (75.7)	154 (51.2)

Absolute prevalence and percentages are reported.

In our sample, 51.8% (*n* = 156/301) scored positively to category #1, whereas 24.3% (*n* = 73/301) and 48.8% (*n* = 147/301) to categories #2 and #3, respectively. Overall, 38.5% (*n* = 116/301) were classified as being high-risk, whereas 61.5% (*n* = 185/301) were low-risk. Table 2 reports general characteristics and medications based on risk categories. High-risk patients had a significantly higher BMI compared to low-risk (high-risk: 28.55 ± 5.14, low-risk: 24.52 ± 3.87; mean difference: 4.03 ± 0.52, *p*-value < 0.001). No differences were observed between smoking and drinking, although a higher proportion of high-risk patients declared these habits and *p*-values were proximal to significance threshold (smoking: *p*-value = 0.052; alcohol: *p*-value = 0.093). A significant difference was observed between gender prevalence, as most high-risk patients were males (in high-risk, males: *n* = 68/116, females: *n* = 48/116; *p*-value < 0.001). Concerning medications, a significantly higher proportion of high-risk patients was found to be taking antiarrhythmic (high-risk: 36/116, low-risk: 29/185, *p*-value: 0.001), antihypertensive (high-risk: 93/116, low-risk: 58/185, *p*-value: < 0.001) and hypoglycemic medications (high-risk: 32/116, low-risk: 23/185, *p*-value: < 0.001). No significant difference was observed for PDE-5 inhibitors, although a higher proportion of at-risk patients was taking medication (high-risk: 9/116, low-risk: 6/185, *p*-value = 0.071; Table 2). At univariate logistic regression analysis, age (OR = 1.03, 95%CI: 1.02–1.05, *p*-value < 0.001), male gender (OR = 1.37, 95%CI: 1.23–1.60, *p*-value < 0.001), and BMI (OR = 1.23, 95%CI: 1.16–1.31, *p*-value < 0.001) were significantly related to patients with poor BQ outcomes. Similarly, those at-risk were more likely to be under antihypertensive (OR = 7.71, 95%CI: 4.35–9.32), *p*-value < 0.001) and diabetes medications (OR = 2.18, 95%CI: 1.28–3.92, *p*-value = 0.024). There were no significant associations between BQ outcomes and assumption of antiarrhythmics (OR = 1.56, 95%CI: 0.32–2.88, *p*-value = 0.133) and PDE-5 inhibitors (OR = 2.51, 95%CI: 0.87–7.25, *p*-value = 0.089; Supplementary Content 2). At multivariate logistic regression analysis, BMI (OR = 1.17, 95%CI: 1.09–1.25, *p*-value < 0.001), as well as antihypertensive (OR = 2.75, 95%CI: 2.02–3.43, *p*-value < 0.001) and diabetes (OR = 1.31, 95%CI: 1.14–1.53, *p*-value = 0.031) medication use were significantly associated with high-risk. Results of the univariate and multivariate regression analyses are presented in Table 3.

**Table 2:** Patients’ characteristics based on risk of sleep apnea syndrome according to the BQ results.

Patient characteristics		High-risk	Low-risk	Difference	*p*-value
BMI (Mean ± SD)		28.55 ± 5.14	24.52 ± 3.87	4.03 ± 0.52^ϕ^	< 0.001
Smoking (%)	Yes	37 (31.9)	42 (22.7)	3.11^η^	0.052
	No	79 (68.1)	143 (77.3)		
Alcohol (%)	>2 times/week	21 (18.1)	22 (11.9)	2.25^η^	0.093
	<2 times/week	95 (81.9)	163 (88.1)		
Gender	Male	68 (58.6)	64 (34.6)	16.71^η^	< 0.001
	Female	48 (41.4)	121 (65.4)		
Medications	Antiarrhythmics	36 (31.0)	29 (15.7)	9.93^η^	0.001
	Antihypertensives	93 (80.2)	58 (31.4)	67.97^η^	< 0.001
	PDE-5 inhibitors	9 (7.8)	6 (3.2)	3.07^η^	0.071
	Hypoglycemics	32 (27.6)	23 (12.4)	10.96^η^	< 0.001

ϕ Student’s t-test.

η Chi-squared test.

**Table 3:** Multivariate regression analysis between single variables and poor BQ outcomes.

Variables		OR (95% CI)	*p*-value	VIF	Box-Tindell transformation
Age		1.00 (0.97–1.02)	0.729	1.17	0.456
Gender	M	1.09 (0.95–1.21)	0.252	1.33	-
	F	1^*^			-
Body mass index		1.17 (1.09–1.25)	< 0.001	1.20	0.312
Alcohol		–		1.42	-
Smoking		–		1.54	-
Medications	Antiarrhythmics	–		1.50	-
	Antihypertensives	2.75 (2.02–3.43)	< 0.001	1.41	-
	PDE-5 inhibitors	–		1.22	-
	Hypoglycemics	1.31 (1.14–1.53)	0.031	1.36	-

OR, odds ratio; CI, confidence interval; VIF, Variance Inflation Factor; BTT, Box-Tindell Transformation (*p*-values are displayed). Estimates not reported due to non-significant results at univariate analysis.

* Reference category.

A sensitivity analysis was performed excluding 119 patients (females = 74/119, 62.18%; mean age = 54.48 ± 13.23) who were referring to the pharmacies for sleep-related breathing concerns or seeking advice for insomnia. Results are presented in Table 4. At univariate analysis, significant associations were found with BMI (OR = 1.16, 95%CI: 1.06–1.25, *p*-value < 0.001), male gender (OR = 1.47, 95%CI: 1.26–1.85, *p*-value = 0.012) and antihypertensive drugs assumption (OR = 3.92, 95%CI: 1.69–9.10, *p*-value = 0.001). Multivariate logistic regression confirmed significant associations with BMI (OR = 1.15, 95%CI: 1.07–1.24, *p*-value < 0.001) and antihypertensive medications use (OR = 2.02, 95%CI: 1.78–3.11, *p*-value = 0.002). Multicollinearity checks showed low impact of each variable in the regression models overall (VIF < 3). A further sensitivity analysis was performed excluding 164 patients (females = 79/164, 48.2%; mean age = 62.48 ± 11.73 years) with known hypertension or adiposity (i.e., BMI > 30). In this subset, the relative prevalence of individuals at-risk for OSA was significantly reduced compared to the overall population (*n* = 13/137, 9.5%, *p*-value < 0.001). Moreover, no additional associations between individual variables and poor BQ outcomes were observed at univariate analysis (Table 5). The assumption of linearity for continuous variables was confirmed in all regression models (*p*-value > 0.05; Tables 3–5).

**Table 4:** Sensitivity analysis after removing patients referring to the pharmacies for insomnia or sleep-related breathing disorders.

Variables		Berlin Questionnaire					
		Univariate		Multivariate		VIF	BTT
		OR (95% CI)	*p*-value	OR (95% CI)	*p*-value		
Age		0.98 (0.96–1.01)	0.192	–		1.05	0.456
Gender	M	1.47 (1.26–1.85)	0.012	1.33 (0.94–1.42)	0.054	1.29	–
	F	1^*^		1^*^			–
Body mass index		1.16 (1.08–1.25)	< 0.001	1.15 (1.07–1.24)	< 0.001	1.08	0.547
Alcohol		1.07 (0.57–1.98)	0.842	–		1.25	–
Smoking		1.54 (0.79–2.97)	0.204	–		1.09	–
Medications	Antiarrhythmics	0.96 (0.52–1.77)	0.894	–		1.08	–
	Antihypertensives	3.92 (1.69–9.10)	< 0.001	2.02 (1.78–3.11)	0.002	1.14	–
	PDE-5 inhibitors	1.19 (0.41–3.51)	0.123	–		1.12	–
	Hypoglycemics	1.13 (0.60–2.15)	0.070	–		1.21	–

OR, odds ratio; CI, confidence interval; VIF, Variance Inflation Factor; BTT, Box-Tindell Transformation (*p*-values are displayed).

* Reference category.

**Table 5:** Sensitivity analysis after removing patients with known hypertension and adiposity (BMI ≥ 30).

Variables		Berlin Questionnaire		
		Univariate		BTT
		OR (95% CI)	*p*-value	
Age		1.03 (0.99–1.07)	0.164	0.211
Gender	M	2.45 (0.77–7.77)	0.128	–
	F	1^*^		–
Body mass index		1.10 (0.93–1.32)	0.240	0.398
Alcohol		0.85 (0.25–2.94)	0.798	–
Smoking		1.45 (0.42–5.08)	0.556	–
Medications	Antiarrhythmics	1.07 (0.12–9.14)	0.954	–
	PDE-5 inhibitors	1.10 (0.17–10.52)	0.845	–
	Hypoglycemics	1.70 (0.34–8.58)	0.522	–

OR, odds ratio; CI, confidence interval. BTT, Box-Tindell Transformation (*p*-values are displayed).

* Reference category.

#### Pittsburgh-sleep quality index

The mean score of each sleep-related subdomain is reported in Table 6. Overall, the mean PSQI derived by the sum of single sleep-domains was 8.57 ± 3.6. This was significantly higher than the mean reported PSQI scores in the healthy Italian population (15) (mean difference 4.57, CI95%: 4.16–4.98, *p*-value < 0.001). Out of the whole sample, 237 patients (*n* = 237/301, 78.7%) scored > 5 and were hence classified as having poor sleep quality. Sixty-four patients (*n* = 64/301, 21.3%) scored ≤ 5 and were defined high-quality sleepers. Patient characteristics according to PSQI outcomes are reported in Table 7. We observed fewer statistically significant differences in general characteristics between patients who were defined poor- or high-quality sleepers according to the PSQI (BMI: mean difference = 0.45 ± 0.67, *p*-value = 0.503; smoking: *p*-value = 0.344; alcohol: *p*-value = 0.568). Similarly, no significant differences were observed between the assumption of specific medications and PSQI scores (antiarrhythmics: *p*-value: 0.401; antihypertensives: *p*-value = 0.432; PDE-5 inhibitors: *p*-value = 0.601; hypoglycemics: *p*-value = 0.481). The relative risk of poor-sleeping according to PSQI outcomes was not associated with baseline demographic characteristics (age: OR = 0.99, 95%CI = 0.97–1.01, *p*-value = 0.237; male gender: OR = 0.68, CI: 0.33–1.02, *p*-value = 0.052; BMI: OR = 1.02, 95%CI = 0.96–1.08, *p*-value = 0.502) nor dependent on the specific medication taken by patients (antiarrhythmics: OR = 0.87, 95%CI = 0.35–1.68, *p*-value: 0.687; antihypertensives: OR = 1.09, 95%CI = 0.63–1.89, *p*-value = 0.755; PDE-5 inhibitors: OR = 1.08, 95%CI = 0.30–3.97, *p*-value = 0.902; hypoglycemics: OR = 1.10, 95%CI = 0.53–2.27, *p*-value = 0.800; Supplementary Content 2). The assumption of linearity of the logit was confirmed for both BMI (*p*-value = 0.394) and age (*p*-value = 0.121). Notably, there was a statistically significant higher prevalence of females among poor-sleepers (in poor-sleepers, males: *n* = 97/237, 40.9%, females: *n* = 140/237, 59.1%; *p*-value: 0.034).

**Table 6:** Results from single domains of the Pittsburgh-sleep quality index.

	Sleep Quality	Sleep Latency	Sleep Duration	Habitual Sleep Efficiency	Sleep Disturbance	Use of sleep medications	Daytime Dysfunction
Mean ± SD (range: 1–3)	1.47 ± 0.74	1.40 ± 0.97	1.30 ± 0.77	0.97 ± 1.11	1.43 ± 0.56	1.01 ± 1.27	0.98 ± 0.83

SD = standard deviation.

**Table 7:** Patients’ characteristics based on sleep quality according to the PSQI results.

Patient characteristics		Poor-sleepers	Good-sleepers	Difference	*p*-value
BMI (Mean ± SD)		26.17 ± 4.80	25.71 ± 4.90	0.45 ± 0.67^ϕ^	0.503
Smoking (%)	Yes	64 (27.0)	15 (23.4)	0.33^η^	0.344
	No	173 (73.0)	49 (76.6)		
Alcohol (%)	>2 times/week	34 (14.3)	9 (14.1)	0.01^η^	0.568
	<2 times/week	203 (85.7)	55 (85.9)		
Gender	Male	97 (40.9)	35 (54.7)	3.88^η^	0.034
	Female	140 (59.1)	29 (45.3)		
Medications	Antiarrhythmics	50 (21.1)	15 (23.1)	0.16^η^	0.401
	Antihypertensives	120 (50.6)	31 (48.4)	0.10^η^	0.432
	PDE-5 inhibitors	12 (5.1)	3 (4.7)	0.02^η^	0.601
	Hypoglycemics	44 (18.6)	11 (17.2)	0.06^η^	0.481

ϕ Student’s *t*-test.

η Chi-squared test.

## Discussion

OSA is a condition with severe systemic consequences and a significant socioeconomic impact (2). The burden of OSA extends beyond the individual, straining healthcare systems, compromising workplace productivity, and affecting overall quality of life. However, its prevalence remains largely underdiagnosed likely due to a combination of diagnostic barriers, misinformation among the general population and the underreporting of symptoms such as snoring and daytime sleepiness (18, 19). Many individuals may not recognize the symptoms of OSA, attributing fatigue and daytime sleepiness to lifestyle factors rather than to an underlying disorder. In recent years, advancements in diagnostic tools, such as at-home cardiorespiratory monitoring and wearable devices, have significantly expanded screening capabilities, making early detection more feasible (20). In this context, community pharmacists play a crucial role, often serving as the first healthcare professionals that patients consult, positioning them as key actors in the diagnostic process (21). The significance of this role is well-established in the literature, to the extent that in 2011, the World Health Organization (WHO), in collaboration with the International Pharmaceutical Federation (FIP), issued the Good Pharmacy Practice guidelines (22), which formally recognize community pharmacists as integral components of a comprehensive healthcare system. Their involvement extends beyond risk identification, encompassing preventive interventions that have been shown to yield tangible health benefits. A systematic review and meta-analysis from Santschi et al., assessing the impact of pharmacist-led care on reducing cardiovascular risk factors, demonstrated that such interventions effectively contribute to reductions in systolic and diastolic blood pressure, cholesterol levels, and smoking prevalence in the general population (23). These findings underscore the essential role of pharmacists in disease prevention and health promotion, further reinforcing their position as essential players in multidisciplinary strategies aimed at early detection and risk mitigation.

Against this background, we conducted a nationwide research to investigate the potential of community pharmacies as a novel frontline setting for OSA screening.

Based on the Berlin Questionnaire results, our data showed that a significant proportion of individuals visiting community pharmacies screened positive for a high risk of sleep-disordered breathing, with an overall screening yield of 38.5%, increasing to 58.6% among males. These figures fall within the higher range of estimates previously reported in the literature (24–28). High-risk patients had a significantly higher BMI and were more frequently male, in line with well-recognized risk factors for OSA (29).

Medication use patterns further reflected this risk profile. Multivariate analysis found that the use of diabetes (OR = 1.31, 95%CI: 1.14–1.53), and particularly antihypertensive (OR = 2.75, 95%CI: 2.02–3.43) medications was significantly higher in high-risk patients, aligning with the recognized association between OSA, metabolic dysfunction, and cardiovascular morbidity (3). In line with this, BMI was the only demographic variable that was still significantly associated with worse BQ outcomes after multivariate adjustment (OR = 1.17, 95%CI: 1.09–1.25, Table 3). Sensitivity analyses confirmed these findings, even though no significant association was still observed with diabetes medications (Tables 4, 5). This evidence reinforces the current recommendations, including those of the American Heart Association (3), which advocate routine OSA screening for patients treated for cardiovascular conditions, given the potential benefits of OSA management on blood pressure control, atrial fibrillation, and heart failure outcomes.

Current guidelines, including those by the US Preventive Services Task Force (USPSTF), conclude that there exists insufficient evidence to recommend widespread OSA screening (7). Our findings, however, suggest that targeted screening may hold particular value, especially in high-risk patient groups, such as those seen in cardiology, and weight management clinics (3).

Community pharmacies may represent an additional and underutilized setting in which such at-risk populations can be efficiently identified. Given their accessibility and trust within the community, pharmacists are well-positioned to engage in early detection efforts, provide patient education on sleep health, and facilitate referrals for further evaluation. Implementing structured screening programs within pharmacies could represent a feasible strategy to facilitate earlier identification of at-risk individuals and support efforts to reduce diagnostic delays, thereby potentially contributing to improved management of patients with coexisting comorbidities. In our analysis of sleep quality, we observed a significantly higher mean PSQI score in this cohort of comorbid patients compared to previously reported values for the healthy Italian population (15). Poor sleep quality is increasingly recognized as a public health concern, impacting cognitive function, emotional well-being, and long-term health (30). However, no associations with demographic characteristics or medications in use emerged from univariate logistic regression analysis (Supplementary Content 2). Similarly, differences in general characteristics and medication use between poor and good sleepers were not significant (Table 7). Also, we observed a higher prevalence of poor sleepers among females, a trend consistently reported in the literature (31). Together, our research highlights the need for a more comprehensive approach to sleep health, considering both physiological and psychosocial factors.

Our study benefits from a large sample size, with the added advantage of the absence of missing data to handle. To reduce the risk of selection bias due to partial overlap between inclusion criteria and questionnaires’ items, we adopted a rigid methodology including multivariate and sensitivity analyses. Moreover, multicollinearity checks and linearity of the logit assessments further mitigated the risk of overestimation of our results. Finally, participation from pharmacies across both urban and rural areas provided valuable geographic diversity, supporting the robustness of our feasibility assessment. However, as this was a convenience sample, the findings should be interpreted as exploratory rather than representative of the national population.

Nonetheless, several limitations should be acknowledged. First, polysomnographic data—the diagnostic gold standard for OSA—was not available. While its inclusion would have substantially strengthened diagnostic accuracy, implementing such an assessment would have posed significant logistic and financial challenges. To address this, we adopted validated screening instruments with high sensitivity for identifying individuals at increased risk for OSA (up to 95% in some studies) (32). Accordingly, our results should be interpreted as reflecting the proportion of subjects at elevated risk rather than the true prevalence of the disorder. This approach aligns with the study’s primary aim of evaluating the feasibility and screening yield of community pharmacies in the pre-diagnostic identification of high-risk individuals, rather than establishing epidemiologic prevalence based on confirmed diagnoses. Second, as a survey-based study, our analysis relies on self-reported data and is thus subject to recall and reporting biases. Third, the absence of a comparison group limits the ability to determine whether pharmacy patients represent a uniquely enriched risk cohort relative to the general population. Furthermore, the inclusion of a proportion of patients seeking help for insomnia or snoring may have modestly overestimated prevalence, although this limit was partially reduced by sensitivity analyses. Finally, we acknowledge that some inclusion criteria (e.g., snoring or sleep-quality concerns) may partially overlap with specific BQ domains, potentially introducing a degree of selection bias and leading to an overestimation of the real prevalence of at-risk patients due to circularity. Multicollinearity checks and multivariate analysis were performed to reduce this risk of bias; nevertheless, further analyses are needed to strengthen those evidences. Taken together, these considerations indicate that while our findings should be interpreted with caution, they nevertheless provide useful insights into the feasibility of pharmacy-based OSA screening and may help guide the design of future studies evaluating the implementation of structured screening programs in this setting.

Future research efforts could focus on enhancing diagnostic accuracy by integrating portable home sleep apnea testing (HSAT) alongside questionnaires. Additionally, establishing follow-up systems for referred patients and incorporating multimodal screening tools, such as nocturnal oximetry or wearable sleep monitors, could improve both sensitivity and specificity while maintaining feasibility in community pharmacy setting. Addressing these topics would strengthen the role of community pharmacists in early OSA detection.

## Conclusion

The burden of OSA on patients and healthcare systems has been well established; however, early detection remains challenging due to limited diagnostic resources and low public awareness. This study provides preliminary evidence supporting the feasibility and potential value of community pharmacies as accessible settings for OSA risk screening. Future research should focus on integrating pharmacist-led screening programs to enhance diagnostic accuracy and patient outcomes. By leveraging the extensive network of community pharmacies, healthcare systems may enhance early identification efforts and promote greater awareness of sleep-disordered breathing within the community.

## Data Availability

The raw data supporting the conclusions of this article will be made available by the authors, without undue reservation.

## Group member of [SIFAC group]

Maria Alessandrello, Giorgia Ascioti, Sara Bianchi, Amedeo Terzo Chiappa, Sara Di Pillo, Beatrice Franceschini, Marisa Gagliardi, Lucia Guida, Giuliana Manara, Camilla Masserini, Giorgia Massetti, Alice Merlin, Marco Oreste Luca Mirra, Matteo Nani, Rosa Sala, Giulia Santucci, Francesca Scarpa, Camilla Segato, Linda Storti.
