# Supplementary material for: Community pharmacies in early detection of obstructive sleep apnea: findings from a nationwide survey
Source: Front Public Health. 2025 Nov 28;13:1712922. doi: 10.3389/fpubh.2025.1712922 (PMC12698553; doi:10.3389/fpubh.2025.1712922)
Supplement: Supplementary file 1 [file Data_Sheet_1.PDF]

Sleep problems and sleep apnea

Questionnaire

Part I: GENERAL QUESTIONNAIRE	
**Q1. Pharmacy Code**<br>_______________	**Q2. Patient Code**<br>_______________
**Q3. Age**<br>_______________	**Q4. Gender**<br>□ Masculine<br>□ Female
**Q5. Height (cm)**<br>_______________	**Q6. Weight (kg)**<br>_______________
**Q7. Smoking habit**<br>□ Yes<br>□ No	**Q8. Alcohol consumption**<br>□ Never<br>□ Rarely<br>□ Occasional (1-2 times a week)<br>□ Regular (3-4 times a week)<br>□ Daily
**Q9. Is the subject taking antiarrhythmic therapy?**<br>□ Yes<br>□ No	**Q10. Is the subject taking antihypertensive therapy?**<br>□ Yes<br>□ No
**Q11. Is the subject taking 5-phosphodiesterase inhibitors (sildenafil and similar)?**<br>□ Yes<br>□ No	**Q12. Is the subject taking hypoglycemic therapy?**<br>□ Yes<br>□ No

**Part II: PITTSBURGH SCALE FOR THE ASSESSMENT OF SLEEP QUALITY**			
The following questions refer to the person's sleep habits during the past month. You will have to indicate the answer that refers to most of the days of the nights of the last month.			
**Q13. During the last month, at what time did you go to bed?**<br>_______________________________ **(hh:mm)**		**Q14. During the last month, how long (expressed in minutes) did it take you to fall asleep each night?**<br>_____________________________________	
**Q15. During the last month, at what time did you wake up in the morning?**<br>_______________________________**(hh:mm)**		**Q16. During the last month, how many hours have you actually slept per night (may be different from the number of hours spent in bed)?**<br>_____________________________**(hh:mm)**	
**Q17. During the last month, how often did you have trouble sleeping because.....**			
**a) You couldn't fall asleep within 30 minutes of going to bed**			
☐ Never during the last month	☐ Less than 1 time per week	☐ 1 or 2 times a week	☐ 3 or more times a week
**b) You woke up in the middle of the night or early in the morning**			
☐ Never during the last month	☐ Less than 1 time per week	☐ 1 or 2 times a week	☐ 3 or more times a week
**c) You had to get up to go to the bathroom**			
☐ Never during the last month	☐ Less than 1 time per week	☐ 1 or 2 times a week	☐ 3 or more times a week
**d) You couldn't breathe well**			
☐ Never during the last month	☐ Less than 1 time per week	☐ 1 or 2 times a week	☐ 3 or more times a week
**e) You coughed or snored loudly**			
☐ Never during the last month	☐ Less than 1 time per week	☐ 1 or 2 times a week	☐ 3 or more times a week
**f) You felt hot**			
☐ Never during the last month	☐ Less than 1 time per week	☐ 1 or 2 times a week	☐ 3 or more times a week
**g) You felt cold**			
☐ Never during the last month	☐ Less than 1 time per week	☐ 1 or 2 times a week	☐ 3 or more times a week
**h) You had bad dreams**			
☐ Never during the last month	☐ Less than 1 time per week	☐ 1 or 2 times a week	☐ 3 or more times a week
**i) You were in pain**			
☐ Never during the last month	☐ Less than 1 time per week	☐ 1 or 2 times a week	☐ 3 or more times a week
**j) For other or more reasons.** You can describe them, including how often you have had trouble sleeping for this reason			
☐ Never during the last month	☐ Less than 1 time per week	☐ 1 or 2 times a week	☐ 3 or more times a week
Reasons____			
**Q18. During the last month, how often did you take medication (prescribed or on your own initiative) to sleep?**			
☐ Never during the last month	☐ Less than 1 time per week	☐ 1 or 2 times a week	☐ 3 or more times a week
**Q19. During the last month, how often have you had trouble staying awake, while, for example, driving, eating, or being employed in some other activity?**			
☐ Never during the last month	☐ Less than 1 time per week	☐ 1 or 2 times a week	☐ 3 or more times a week
**Q20. During the last month, how problematic has it been for you to maintain your enthusiasm for the things you were doing?**			
☐ Never during the last month	☐ Less than 1 time per week	☐ ☐ 1 or 2 times a week	☐ 3 or more times a week
**Q21. During the last month, how would you rate your sleep quality?**			

▯ Very good	▯ Pretty good	▯ Pretty bad	▯ Very bad

Part III: BERLIN QUESTIONNAIRE

CATEGORY 1	
**Q22. Do you usually snore?**<br>□ At. Yes<br>□ B. No<br>□ C. I don't know	**Q23. If you snore, your snoring is:**<br>□ At. Slightly louder than your breathing<br>□ B. Louder than other people speaking<br>□ C. Louder than speech<br>□ Q. Very high – can be heard in adjacent rooms
**Q24. How often do you snore?**<br>□ At. Almost every day<br>□ B. 3-4 times a week<br>□ C. 1-2 times a week<br>□ Q. 1-2 times a month<br>□ And. Never or almost never email	**Q25. Has your snoring ever affected other people?**<br>□ At. Yes<br>□ B. No<br>□ C. I don't know
**Q26. Has anyone noticed that you stop breathing during sleep?**<br>□ At. Almost every day<br>□ B. 3-4 times a week<br>□ C. 1-2 times a week<br>□ Q. 1-2 times a month<br>□ And. Never or almost never email	
**CATEGORY 2**	
**Q27. How often do you feel tired or fatigued after a night's sleep?**<br>□ At. Almost every day<br>□ B. 3-4 times a week<br>□ C. 1-2 times a week<br>□ Q. 1-2 times a month<br>□ And. Never or almost never email	**Q28. During the day, do you feel tired or fatigued ?**<br>□ At. Almost every day<br>□ B. 3-4 times a week<br>□ C. 1-2 times a week<br>□ Q. 1-2 times a month<br>□ And. Never or almost never email
**Q29. Have you ever dozed off or fallen asleep while driving a vehicle?**<br>□ At. Yes<br>□ B. No	**Q30. If so, how often does the problem occur?**<br>□ At. Almost every day<br>□ B. 3-4 times a week<br>□ C. 1-2 times a week<br>□ Q. 1-2 times a month<br>□ And. Never or almost never email
**CATEGORY 3**	
**Q31. Do you have high blood pressure?**<br>□ Yes<br>□ No<br>□ I don't know	
