# Supplementary material for: Community pharmacies in early detection of obstructive sleep apnea: findings from a nationwide survey
Source: Front Public Health. 2025 Nov 28;13:1712922. doi: 10.3389/fpubh.2025.1712922 (PMC12698553; doi:10.3389/fpubh.2025.1712922)
Supplement: Supplementary file 2 [file Data_Sheet_2.PDF]

Supplementary content 2. Univariate regression analysis between individual variables and poor questionnaire outcomes. OR = unadjusted Odds Ratio; CI: confidence interval. * Reference category.

		Berlin Questionnaire		Pittsburgh-sleep quality index	
		OR (95% CI)	p-value	OR (95% CI)	p-value
Age		1.03 (1.02 – 1.05)	< 0.001	0.99 (0.97 – 1.01)	0.237
Gender	M	1.37 (1.23 – 1.60)	< 0.001	0.68 (0.33 – 1.02)	0.052
	F	1*		1*	
Body mass index		1.23 (1.16 – 1.31)	< 0.001	1.02 (0.96 – 1.08)	0.502
Alcohol		0.94 (0.57 – 1.56)	0.810	1.17 (0.65 – 2.11)	0.613
Smoking		1.59 (0.95 – 2.68)	0.079	1.21 (0.63 – 2.31)	0.565
Medications	Antiarrhythmics	1.56 (0.32 – 2.88)	0.133	0.87 (0.35 – 1.68)	0.687
	Antihypertensives	7.71 (4.35 – 9.32)	< 0.001	1.09 (0.63 – 1.89)	0.755
	PDE-5 inhibitors	2.51 (0.87 – 7.25)	0.089	1.08 (0.30 – 3.97)	0.902
	Hypoglycemics	2.18 (1.28 – 3.92)	0.024	1.10 (0.53 – 2.27)	0.800
